# Socio-demographic predictors of health and environmental co-benefit behaviours for climate change mitigation in urban China

**DOI:** 10.1371/journal.pone.0188661

**Published:** 2017-11-27

**Authors:** Emily Ying Yang Chan, Susan Shuxin Wang, Janice Ying-en Ho, Zhe Huang, Sida Liu, Chunlan Guo

**Affiliations:** 1 Collaborating Centre for Oxford University and CUHK for Disaster and Medical Humanitarian Response (CCOUC), The Chinese University of Hong Kong, Shatin, Hong Kong SAR, China; 2 The Jockey Club School of Public Health and Primary Care, Faculty of Medicine, The Chinese University of Hong Kong, Shatin, Hong Kong SAR, China; 3 Nuffield Department of Medicine, University of Oxford, Oxford, United Kingdom; TNO, NETHERLANDS

## Abstract

**Objective:**

This study aims to examine the patterns and socio-demographic predictors of health and environmental co-benefit behaviours that support climate change mitigation in a densely populated Asian metropolis—Hong Kong.

**Methods:**

A population-based, stratified and cross-sectional random digit dialling telephone survey study was conducted between January and February 2016, among the Cantonese-speaking population aged 15 and above in Hong Kong. Socio-demographic data and the self-reported practice of 10 different co-benefit behaviours were solicited. Ethics approval and participant’s verbal consent were sought.

**Findings:**

The study sample consisted of 1,017 respondents (response rate: 63.6%) were comparable to the age, gender and geographical distributions of the Hong Kong population found in the latest 2011 Hong Kong Population Census. Among the co-benefit behaviours, using less packaging and disposable shopping bags were practiced in the highest frequency (70.1%). However, four behaviours were found to have never been practiced by more than half of the respondents, including bringing personal eating utensils when dining in restaurants or small eateries, showering less than five minutes, having one vegetarian meal a week, and buying more organic food. Results of multivariable logistic regression showed that frequency of practicing co-benefit behaviours were consistently associated with gender and age.

**Conclusion:**

Urban residents in Hong Kong do not engage in the practice of co-benefit behaviours in a uniform way. In general, females and older people are more likely to adopt co-benefit behaviours in their daily lives. Further research to assess the knowledge and attitudes of the population towards these co-benefit behaviours will provide support to relevant climate change mitigation policies and education programmes.

## Introduction

Climate change is known to pose risks to human health [1,2]. Through rising temperature, increasing variability in precipitation, rising sea levels, and a growing number of extreme weather events, climate change will not only exacerbate existing human health problems, but also affect people’s livelihoods and damage critical health infrastructure [1,2]. As human behaviour is regarded as a primary cause of climate change [1,2], modifying our behaviour has been confirmed as one of the efficient and low-cost methods to mitigate climate change [2,3].

Climate change and human health are so closely linked that many mitigation measures naturally promote public health and environmental benefits at the same time, as captured in the concept of co-benefits. As defined by Intergovernmental Panel on Climate Change (IPCC) and World Health Organization (WHO), co-benefits involve “a climate change adaptation or mitigation strategy which has additional, positive effects on health or other areas” [4] and “health gains from strategies that are directed primarily at climate change, and mitigation of climate change from well-chosen policies for health advancement” [5], respectively. While co-benefits can be applied across a variety of sectors, the co-benefit behaviours this study investigates refer specifically to the behaviours that have positive effects on health and environment. Highlighting the health and environmental co-benefits could provide more motivation and justification for climate change mitigation actions than focusing only on the environmental benefits [6–8].

The significant effect of individual lifestyles on both environmental change and health has drawn rapidly increasing attention in the research community recently [9]. For instance, augmented physical activity as a result of walking and cycling to destinations instead of driving will not only reduce carbon emissions and air pollution, but also lower the risk of chronic diseases [10]. Previous studies have provided evidence of co-benefits in areas of active travel (walking and cycling) [11], public transport [12], food-related behaviours (vegetarian lifestyle, organic and locally-sourced food, and plate waste) [13], household energy use and waste management [14]. In the United States, five categories of household actions can provide a behavioural wedge to rapidly reduce greenhouse gas (GHG) emissions: i) home weatherization and upgrades for heating and cooling equipment, ii) energy-efficient vehicles and appliances, iii) equipment maintenance, iv) equipment adjustment, and v) daily energy use behaviours (e.g. standby electricity and driving behaviour) [15]. However, co-benefits need to be adapted locally, as the benefits are largely contextual and affected by local practices, culture, lifestyle habits and so on. [16]. In addition, there is currently a dearth of evidence on the patterns and predictors of such co-benefit practices. More evidence is urgently needed to understand the associations between the practices of healthy behaviours and demographical factors [17], or between environmental friendly behaviours and demographic factors [18,19], particularly in a densely populated urban context. This study investigates the patterns of 10 different co-benefit behaviours relevant to an Asian urban community, and examines the relationship between demographics and the frequency of practicing co-benefit behaviours. The findings will provide valuable insight for tailoring behavioural interventions for different population subgroups.

## Materials and methods

### Study design

A population-based, stratified and cross-sectional random digit dialling telephone survey was conducted between 28 January and 4 February 2016. The survey was conducted in Cantonese language, as 95.8% of the Hong Kong population are Cantonese speakers or able to use Cantonese [20]. Over 95% of Hong Kong households have a land-line telephone [21], which enhances the validity of using the telephone survey methodology to conduct a representative study of the general population. The study population was the non-institutionalized population aged 15 years or above residing in Hong Kong, including residents holding valid work or study visas. Exclusion criteria included i) non-Cantonese-speaking respondents; ii) children under the age of 15; iii) overseas visitors holding tourist visas to Hong Kong or two-way permit holders from mainland China; and iv) those unable to be interviewed due to medical reasons.

For the sample size calculation, we assumed the prevalence of co-benefit behaviours is 50%, which was a conservative hypothesis. A sample size of 784 participants was calculated with a 3.5% margin of error and 95% confidence interval. To account for potential missing data and increase the modelling flexibility, 1,017 participants were recruited. In addition, quota sampling was used to ensure the demographic representation of the general population in Hong Kong in terms of age, gender, and district of residence.

### Ethics approval and participant consent

Ethics approval and consent procedure of the study was reviewed and obtained from the Survey and Behavioural Research Ethics Committee at The Chinese University of Hong Kong (dated on 13 January 2016). Verbal consent was obtained from each participant in the beginning of the study. Each participant was required to indicate their willingness to participate in the survey and have the interview audially recorded. The interview was stopped immediately when the participant required to exit the survey. Similar to previous research studies with the same telephone study methodology that target general population [22–25], no additional consent was required for age 15–17 by the university research ethics committee and thus additional consent was not sought from participants aged 15–17 in this study.

### Instrument

A self-reported questionnaire was designed for data collection, which included information on the general socio-demographic background and practices of 10 different co-benefit behaviours of a respondent, as seen in S1 File. The study was one of the key components of a larger climate change, extreme temperatures, and health survey [26]. The socio-demographic background section comprised of questions on gender, age, district of residence, marital status, education attainment, monthly household income, home ownership status, and type of housing. The 10 co-benefit behaviours were enquired through a common question: “In the past year, did you engage in the following lifestyle habits?” Behavioural frequencies of practicing 10 co-benefit behaviours were reported on a 5-point Likert scale: Never practiced nor considered, Never practiced but considered, Occasionally practiced, Practiced at least once a week, and Practiced daily (see S1 File).

Table 1 includes the specific co-benefit behaviours, their linkages to health and environment, and their specific benefits in these areas. The 10 co-benefit behaviours were chosen to address both the practices supported by scientific evidence (see Table 1) as well as local community initiatives, such as showering less than five minutes every day [27]. Some co-benefit behaviours have been well-studied in high-income countries: travel behaviours [11,12], food-related behaviours [13], household energy use and waste management [14]. However, in a densely populated Asian metropolis context like Hong Kong, the co-benefit behaviours need to be adjusted to the local context. For example, promoting the use of public transport is not as relevant in the territory because the rate of private car ownership in Hong Kong is low (0.07 car per person, compared with 0.46 in the United States and 0.41 in Japan) [28,29] and a majority of the residents use public transportation daily. In a similar vein, all the co-benefit behaviours have been chosen in this study for their local relevance in revealing the pattern of behaviours with significant health and environmental impacts among Hong Kong residents.

**Table 1:** Health and environmental benefits of 10 co-benefit behaviours^a^.

Category	Behaviour	Linkage	Health benefits	Environmental benefits
**Active travel**	Walk/cycle more	Reduce the use of motorized transportation, air pollutant emissions (e.g. particulate matter, ozone, volatile organic compounds), physical inactivity, and risk factors (e.g. obesity) of non-communicable diseases [30]	Reduce the risks of chronic diseases (e.g. cardiovascular diseases, diabetes, cancers), premature death, respiratory symptoms and illnesses (e.g. asthma, lung cancer), injuries from traffic accidents, depression, and mental health problems[30]	Improve air quality, and reduce the operation of internal combustion engines and the emissions of greenhouse gases (GHG) and smog-forming VOCs and NO_x_ [31]
**Dietary-related behaviours**	Buy more organic food	Reduce the exposure to additives, chemical fertilisers or pesticides (e.g. insecticides, fungicides, rodenticides, pediculicides, and biocides) via inhalation, ingestion, dermal contact, or across the placenta [32]	Reduce the risks of allergies, hay fever, cancer development (e.g. leukaemia), neurodevelopmental delays in children, and triggers for multiple chemical sensitivity [32]	Improve water and soil quality, and reduce soil degradation due to pesticides and the development of resistance in insects [33]
	Consume less meat	Reduce GHGs produced by ruminant livestock (e.g. cows) and over-consumption of red meat which usually contains more saturated fats [34]	Reduce the risks of colorectal cancer, cardiovascular diseases, diabetes, and lung cancer potentially associated moderately with exposure to high temperature-cooking [34]	Reduce nitrogen and GHG emissions, and decrease land scarcity through less demand for cropland to grow animal feed [34,35]
	Have one vegetarian meal a week	Reduce over-consumption of food with high-fat content (e.g. saturated fats, trans-fats) [36]	Reduce the risk of diseases (e.g. constipation, diverticular disease, gallstones and appendicitis) and obesity, thereby lowering the risk of chronic diseases (e.g. coronary heart diseases) [36]	Reduce GHG emissions, and conserve water and energy since the vegetarian diet requires less water, primary energy, fertilizers and pesticides than the non-vegetarian diet [37]
**Household conservation**	Use less electricity	Alleviate air pollution from fossil fuel power plants (i.e. those burning coal, petroleum and natural gas) [38]	Reduce the risks of stroke, heart disease, lung cancer, and chronic lower respiratory tract diseases [7]	Reduce GHG emissions, air pollution, and coal combustion waste, which could contaminate groundwater and soil if disposed improperly [38,39]
	Use less air conditioning (AC)	Improve indoor air quality (IAQ) and increase indoor air exchange rate, air movement and ventilation with open windows [40]	Reduce the concentrations of indoor particle pollutants and VOCs, prevalence of sick building syndrome (SBS), and the risk of respiratory allergy [40]	Reduce the release of anthropogenic heat, prevalence of urban heat island effect, and pollutants released from refrigerants [41]
	Shower less than five minutes every day	Conserve limited water resources by reducing average household water consumption [42]	Secure the local availability of clean water for drinking, cooking, and personal hygiene to reduce the risks of infectious diseases transmitted by water, food, and contact [43]	Reduce the impacts of wastewater discharges on environmental water quality and conserve biodiversity [42,44]
**Waste management**	Use less packaging and fewer disposable shopping bags	Reduce plastic waste and migration of chemicals from plastic bags and packaging materials [45]	Reduce the risks of breast cancer and other disruptions to human reproductive functions potentially related to exposure to chemicals found in plastics (e.g. Bisphenol A) [46,47]	Reduce the landfill burden, plastic debris in the marine or terrestrial environments, and GHG emissions from plastic production and combustion [48,49]
	Bring personal eating utensils when dining in restaurants or small eateries	Reduce plastic waste and exposure to harmful chemicals, and increase protection of hygiene [48,50–52]	Protect personal hygiene and reduce potential risk of breast cancer, obesity, immune disorders, early puberty, reproductive harm and other health disorders due to endocrine disruption from Bisphenol A [50–52]	Reduce the landfill burden, plastic debris in the marine or terrestrial environments, and GHG emissions from plastic production and combustion [48,49]
	Separate household waste	Reduce the amount of waste sent to landfills, particularly household hazardous waste [53,54]	Reduce the risks of congenital anomalies, reproductive disorders, and the risk of cancer development [53]	Increase the amount of material recovery and reduce the landfill burden, GHG emissions from primary material production, and leachate/migration of hazardous chemicals and other emissions (e.g. volatile organic compounds, particulate matter) into the surrounding environment of landfills [48,54,55]

^a^ The term Health and Environmental Co-benefit Behaviours can be used in further discussions.

The survey questionnaire was pilot-tested and revised in December 2015. Fifty-two samples were collected through a population-based, stratified and cross-sectional telephone survey, using random digit dialling method. The pilot study also adopted a quota sampling method to ensure the sample’s representativeness of the general population in terms of age, gender and district of residence. Quota sampling was also used in the main study. After the pilot test, additional questions related to the behaviours of showering less than five minutes every day, consuming less meat, and having one vegetarian meal a week were included to capture a more comprehensive picture of practicing health and environmental co-benefits behaviours. A binary scale of practicing co-benefit behaviours or not was modified into a 5-point Likert scale in the main survey tool to obtain a more finely differentiated frequency of practicing these behaviours.

### Data collection

The telephone survey was conducted from 28 January to 4 February 2016. The Random Digit Dialling (RDD) method was used for each of Hong Kong’s 18 districts to generate a randomly selected representative sample. Telephone calls were made during weekday evenings (6:30pm to 10:00pm, Monday to Friday) and during daytime on the weekends (Saturday and Sunday) to avoid an under-representation of the working population. A participant was selected from each of the contacted households through the “last birthday method”. An eligible family member (defined as a Cantonese-speaking Hong Kong resident over the age of 15 without hearing impairment) who has passed the birthday most recently was invited to participate in the study. At least five attempts were made in different time slots to reach a household by dialling a number before that number was considered invalid. All telephone interviews were conducted by trained interviewers in Cantonese. Each interview took approximately 15–20 minutes to complete.

### Data analysis

Descriptive statistics on the socio-demographic variables and frequencies of practicing the 10 co-benefit behaviours were reported. A backward-stepwise (likelihood ratio) multivariable logistic regression was performed to investigate the association between socio-demographic variables and the adoption of co-benefit behaviours. Respondents were excluded from a regression analysis if they had refused to answer the pertaining co-benefit behaviour question. All statistical analyses were performed using IBM SPSS Statistics 21 for Windows at a significance level of ɑ = 0.05.

## Results

### Participants and demographics

A total of 3,500 telephone numbers were dialled, of which 1,598 calls were responded to by eligible persons. Among them, 1,125 eligible respondents agreed to participate and gave verbal consent, and 1,017 respondents successfully completed the questionnaire, with a response rate of 63.6% (1017/1598). As shown in Table 2, the sample was representative of the population characteristics found in the latest 2011 Hong Kong Population Census in terms of the distributions of gender, age, district, education level, marital status, and home ownership status.

**Table 2:** Socio-demographic characteristics of study participants and the Hong Kong general population.

Demographics	Sample participants		2011 Hong Kong Population Census	Sample vs. Census p-value ^a^
	n	%		
**Gender**	1017			
Male	437	43.0%	46.0%	0.670
Female	580	57.0%	54.0%	
**Age**	1017			
15–24	126	12.3%	14.0%	0.824
24–44	315	31.0%	35.5%	
45–64	384	37.8%	35.4%	
≥65	192	18.9%	15.1%	
**District**	1015			
Hong Kong Island	182	17.9%	18.0%	0.981
Kowloon	315	31.0%	29.8%	
New Territories	518	51.0%	52.2%	
**Education**	1015			
Primary or below	137	13.5%	22.7%	0.147
Secondary	501	49.4%	50.0%	
Post-secondary or above	377	37.1%	27.3%	
**Marital status**	1012			
Single	410	40.5%	42.2%	0.807
Married	602	59.5%	57.8%	
**Monthly household income**	945			
<20,000	295	31.2%	47.5%	0.033
20,000–39999	333	35.2%	29.0%	
≥40000	317	33.5%	23.5%	
**Home ownership**	1000			
Owned	629	62.9%	52.1%	0.122
Rent	371	37.1%	47.9%	
**Housing type**	1012			
Public housing	336	33.2%	30.3%	0.946
Subsidized home ownership housing	173	17.1%	15.9%	
Private permanent housing	486	48.0%	52.3%	
Others	17	1.7%	1.5%	

^a^ Chi-square test was used to measure the overall difference in demographic proportions between this study and the 2011 Hong Kong Population Census [20]. *p*- value <0.05 indicates a significant difference.

The participants comprised of 437 males and 580 females. Over half of the participants were 45 years old or above (56.7%). Around half of the participants were from the New Territories district. Nearly two-thirds of them had received only a secondary education or below (62.9%). Approximately 60% of the participants were married and one-third of them had a household income of less than HKD 20,000 per month (USD 2,564). While two-thirds owned their residence (62.9%), one-third lived in public housing (33.2%) (see Table 2).

### Descriptive analysis

As shown in Table 3, the frequencies of practicing different types of co-benefit behaviours varied greatly. The most frequently practiced co-benefit behaviour was Use less packaging and fewer disposable shopping bags, which was practiced daily among 70.1% of the population. Several co-benefit behaviours had a daily practice among approximately 50% of the population, including Walk/cycle more (54.8%), Separate household waste (50.2%), Use less electricity (48.3%), and Use less AC (44.1%). Those that were practiced daily by around 30% of the population included Consume less meat (33.3%) and Shower less than five minutes every day (23.7%). Behaviours that were practiced daily by less than 10% of the population were Have one vegetarian meal a week (5.8%), Buy more organic food (4.3%), and Bring personal eating utensils when dining in restaurants or small eateries (4.0%).

**Table 3:** The frequencies of practicing co-benefit behaviours among Hong Kong population.

Category	Behaviour	Daily	At least once a week	Occasionally	Never practiced but considered	Never practiced nor considered	Sample size (n)
**Active travel**	Walk/cycle more	54.8%	22.0%	5.7%	8.8%	8.7%	1013
**Dietary-related behaviours**	Buy more organic food	4.3%	15.6%	20.6%	23.4%	36.2%	1009
	Consume less meat ^a^	33.3%	24.3%	9.0%	7.7%	25.6%	975
	Have one vegetarian meal a week ^a^	5.8%	24.6%	8.5%	12.1%	49.0%	978
**Household consumption**	Use less electricity	48.3%	14.0%	9.3%	14.6%	13.8%	1007
	Use less AC	44.1%	22.9%	12.2%	11.6%	9.2%	986
	Shower less than five minutes every day	23.7%	10.0%	4.2%	13.3%	48.8%	1004
**Waste management**	Use less packaging and fewer disposable shopping bags	70.1%	19.7%	5.8%	1.4%	3.0%	1014
	Bring personal eating utensils when dining in restaurants or small eateries	4.0%	5.0%	5.7%	14.9%	70.3%	1015
	Separate household waste	50.2%	11.5%	6.2%	14.8%	17.2%	1011

^a^ These behaviours excluded those who self-reported to be vegetarians.

### Multivariable logistic regression

The results of multivariable logistic regression with backward stepwise elimination indicated that the adoption of different types of co-benefit behaviours were associated with different socio-demographic variables, as indicated in Table 4. In general, females were more inclined to practice co-benefit behaviours, especially Use less packaging and fewer disposable shopping bags (AOR = 6.34, 95% CI: 2.75–14.60), Have one vegetarian meal a week (AOR = 2.39, 95% CI: 1.79–3.18), Buy more organic food (AOR = 2.19, 95% CI: 1.65–2.91), Consume less meat (AOR = 2.14, 95% CI: 1.60–2.87), Separate household waste (AOR = 1.99, 95% CI: 1.49–2.66), Use less electricity (AOR = 1.59, 95% CI: 1.19–2.14), Use less AC (AOR = 1.54, 95% CI: 1.11–2.13), and Bring personal eating utensils when dining in restaurants or small eateries (AOR = 1.53, 95% CI: 1.05–2.23) (see Table 4).

**Table 4:** Multivariable logistic regression results of co-benefit behaviours and demographics in Hong Kong.

Demographics	Active travel	Dietary			Household consumption			Waste management		
	Walk/cycle more (N = 917)	Buy more organic food (N = 917)	Consume less meat (N = 886)	Have one veg. meal a week (N = 887)	Use less electricity (N = 915)	Use less AC (N = 898)	Shower less than 5 min every day (N = 910)	Use less packaging and… bags (N = 919)	Bring personal eating utensils… (N = 919)	Separate household waste (N = 917)
	Adjusted Odds Ratio (95% Confidence Interval)									
**Gender**										
Male		1	1	1	1	1		1	1	1
Female		2.19(1.65–2.91)***	2.14(1.60–2.87)***	2.39(1.79–3.18)***	1.59(1.19–2.14)**	1.54(1.11–2.13)*		6.34(2.75–14.60)***	1.53(1.05–2.23)*	1.99(1.49–2.66)***
**Age**										
15–24		1	1	1	1		1			1
24–44		2.57(1.50–4.39)***	1.37(0.88–2.14)	1.57(0.95–2.59)	1.37(0.87–2.15)		1.34(0.83–2.17)			2.66(1.69–4.18)***
45–64		4.30(2.54–7.29)***	3.44(2.18–5.43)***	2.36(1.37–4.05)**	1.73(1.11–2.72)*		2.21(1.39–3.51)***			2.97(1.90–4.64)***
≥65		3.34(1.83–6.10)***	3.32(1.96–5.63)***	1.88(1.05–3.35)*	2.27(1.33–3.86)**		1.81(1.08–3.04)*			2.80(1.69–4.66)***
**Education**										
Primary or below									1	
Secondary									1.75(0.87–3.54)	
Post-secondary or above									2.39(1.18–4.84)*	
**Marital status**										
Single	1			1						
Married	1.96(1.38–2.79)***			0.65(0.47–0.91)*						
**Household income**										
<20,000		1								
20,000–39999		1.20(0.84–1.73)								
≥40000		1.66(1.15–2.40)**								
**Housing type**										
Public housing	1					1				1
Subsidized home ownership housing	1.57(0.87–2.82)					1.03(0.61–1.72)				2.19(1.40–3.42)***
Private permanent housing	0.75(0.51–1.10)					0.62(0.43–0.90)*				1.46(1.07–2.01)*
**Predicted percentage**	83.0%	62.7%	68.1%	62.6%	71.7%	79.8%	62.3%	95.9%	84.7%	69.1%
**-2 Log likelihood**	814.28	1160.25	1050.49	1129.65	1068.93	887.72	1189.61	274.37	776.46	1089.08
**Nagelkerke R**^**2**^	0.040	0.106	0.116	0.078	0.033	0.026	0.024	0.154	0.021	0.097

The Backward Stepwise (Likelihood Ratio) method was adopted in the multivariable logistic regression analyses and the insignificant independent variables were deleted from the final model of each co-benefit behaviour. District was adjusted for in the modelling but did not demonstrate any significant associations with the co-benefit behaviour outcomes in this study.

*** *p*≤0.001;

** *p* ≤0.01;

* *p*≤0.05.

Older people, both those between 45 and 64 and those 65 and above, were more likely to practice co-benefit behaviours daily when compared with younger people. Associations with older age were found in the following co-benefit behaviours: Buy more organic food (age 45–64: AOR = 4.30, 95% CI: 2.54–7.29; age≥65: AOR = 3.34, 95% CI:1.83–6.10), Consume less meat (age 45–64: AOR = 3.44, 95% CI: 2.18–5.43; age≥65: AOR = 3.32, 95% CI: 1.96–5.63), Have one vegetarian meal a week (age 45–64: AOR = 2.36, 95% CI: 1.37–4.05; age ≥65: AOR = 1.88, 95% CI: 1.05–3.35), Use less electricity (age 45–64: AOR = 1.73, 95% CI: 1.11–2.72; age≥65: AOR = 2.27, 95% CI: 1.33–3.86), Shower less than five minutes every day (age 45–64: AOR = 2.21, 95% CI: 1.39–3.51; age≥65: AOR = 1.81, 95% CI: 1.08–3.04), and Separate household waste (age 45–64: AOR = 2.97, 95% CI: 1.90–4.64; age≥65: AOR = 2.80, 95% CI: 1.69–4.66).

Among the 10 co-benefit behaviours, education level was significantly associated with only bringing personal eating utensils when dining in restaurants or small eateries. The participants with post-secondary education level or above had 139% higher frequency of bringing their personal eating utensils than those who obtained primary or below education (AOR = 2.39, 95% CI: 1.18–4.84).

Regarding the other socio-demographic predictors, marital status was significantly associated with active travel and having one vegetarian meal a week. Married participants were 96% more inclined to walk/cycle more in daily life (AOR = 1.96, 95% CI: 1.38–2.79) but 35% less intended to have one vegetarian meal a week (AOR = 0.65, 95% CI: 0.47–0.91). Household income was significantly associated only with buying more organic food. Participants with household incomes of HKD 40,000 or above per month were at least 66% more likely to buy more organic food daily (AOR = 1.66, 95% CI: 1.15–2.40). Compared with those living in public housing, respondents who lived in private permanent housing were less likely to use less AC (AOR = 0.62, 95% CI: 0.43–0.90) but more willing to separate household waste (AOR = 1.46, 95% CI: 1.07–2.01), whereas those who lived in subsidized home ownership housing were also more willing to separate household waste (AOR = 2.19, 95% CI: 1.40–3.42).

In summary, among the 10 co-benefit behaviours, using less packaging and disposable shopping bags was practiced daily by the highest proportion of people (70.1%). However, four behaviours were found to have been practiced by less than half of the population, including bringing personal eating utensils when dining in restaurants or small eateries, showering less than five minutes, having one vegetarian meal a week and buying more organic food. Multivariable logistic regression results showed that practicing co-benefit behaviours were consistently associated with gender and age.

## Discussion

### Diverse patterns of co-benefit behaviours

As indicated in the findings, urban population in Asia showed a diverse pattern in the practice of health and environmental co-benefit behaviours in their daily life. Using less packaging and fewer disposable shopping bags received the highest frequency in daily practice (70.1%), reflecting success of the 2015 Plastic Shopping Bag Levy Scheme, a regulatory policy that requires a charge of HKD 0.5 (USD 0.06) for a plastic shopping bag [56]. However, behaviours within the same categories also experienced a significant variation in self-reported practice frequency. The practices of having one vegetarian meal a week, bringing personal eating utensils when dining in restaurants or small eateries, and buying more organic food had a substantially lower proportion of daily practice than other behaviours in the same categories (4.0%-5.8%). Although the behaviours of consuming less meat and having one vegetarian meal a week bear similar incentives as well as health and environmental benefits, the proportions of daily practice among the population differed significantly (daily practice of Consume less meat (33.3%) vs. Have one vegetarian meal a week (5.8%), χ^2^(4) = 27.681, *p*<0.001). This may be attributed to the perception and ease of the behaviour among the respondents, as it may be easier to reduce practicing a behaviour than it is to schedule or regulate a specific dietary change. A similar situation can be found for the behaviours of using less packaging and fewer disposable shopping bags and bringing personal eating utensils when dining in restaurants or small eateries, whereby reducing the practice of a current behaviour (albeit with considerable policy support) was found among a significantly higher proportion of people (70.1%) than instilling the practice of a non-normative behaviour (4.0%) (χ^2^(4) = 140.685, *p*<0.001). Interestingly, in both cases, the patterns of socio-demographic associations were similar between the two behaviours despite differences in proportion of daily practice. In the case of buying organic food, the low proportion of daily practice (4.3%) can be attributed to a smaller share in the overall food market, complications around certification and labelling, and higher prices [57], as supported by the findings of an association with higher monthly income in this research.

### Gender differences in the practice of co-benefit behaviours

Females consistently performed better than males across different co-benefit behaviours, which is consistent with the findings of previous studies on the gender associations of environmental friendly behaviours [18]. This study found that women are more inclined to use less electricity and AC, use less packaging and few disposable shopping bags, bring personal eating utensils when dining in restaurants or small eateries, buy more organic food, and separate household waste. The associations found could potentially be due to the attitude and practices arising from traditional gender roles, where women are generally more involved in household affairs (e.g. shopping and cooking) than men [58]. Additionally, women were found to be more likely to have one vegetarian meal a week and consume less meat. The lower proportion of practicing vegetarian-related behaviours by men could be associated with perceptions of meat consumption as a sign of masculinity [59–62]. Future promotion initiatives should confront traditional gender roles and stereotypes.

### Age cohort differences in practicing co-benefit behaviours

Contrary to a popular belief, our study findings demonstrated that younger generations do not necessarily perform better in behaviours that benefit health and the environment. Instead, older people (age 45 or above) were more likely than younger people (age 44 or below) to practice co-benefit behaviours daily, especially for all dietary-related behaviours, reducing electricity and water usage, as well as separating household waste. These findings reveal a pattern similar to a meta-analysis of the relationship between age and pro-environmental variables, where older age was associated with more conservation behaviours (i.e. reducing use, avoiding waste, reusing, repurposing, and recycling) [19]. This may be attributable to a higher concern for frugality and conscientious behaviour among older people [19]. In this study, those in the age cohort of 45 or above were born between 1930s and 1960s, right around the period of wartime and post-war rapid industrialization in Hong Kong, thus quite possibly growing up in a lifestyle of limited resources and conservation habits. Therefore, future promotion of conservation-related co-benefit behaviours should target and educate younger generations.

### Variation of co-benefit practices with income and real estate ownership

Income and real estate ownership had diverse associations with the practice of different co-benefit behaviours. Those with higher household income were more likely to buy more organic food. A possible explanation could be that those with lower household income had a lower proportion of disposable income available for purchasing higher-priced organic food. On the other hand, respondents who lived in private permanent housing were less likely to use less AC, while both they and those living in subsidized housing were more likely to separate household waste when compared with public housing residents. Generally, the residents in subsidized housing have higher monthly income than the group who live in public housing, but lower than the group in private housing.

## Limitations

This paper used a population-based, stratified and cross-sectional random digit dialling telephone survey to collect data on co-benefit behaviours in the general population of an urban Asian metropolis. The cross-sectional study design can only demonstrate associations between patterns and social-demographic predictors, as causation cannot be attributed to the findings. In addition, data collection through the telephone survey was based on self-reported information, which might be subjected to reporting bias as well as imprecisions in the measurement of each co-benefit behaviour of interest. Moreover, with the finite amount of time in each telephone interview, this study could only collect data based on a limited number of questions and this restricted our ability to examine each of the behavioural patterns in detail. Furthermore, although the scientific relationships between health and environmental co-benefits of the studied health messages in this paper are based on sound theoretical deduction, and various published studies have demonstrated associations between the behaviour patterns and health outcomes, more scientific investigations are needed to quantify the relationship between the health outcomes and co-benefit behaviours, and to explore what might be other non-knowledge-based factors (such as environmental context) and if the level of behavioural adoption might affect actual health outcomes.

### Policy implications

The local government has previously implemented a series of measures to promote co-benefit behaviours among the Hong Kong population. However, as supported by our study findings, the impact of these measures varied. For example, the Plastic Shopping Bag Levy Scheme implemented in 2015, which charges HKD 0.50 (USD0.06) per plastic shopping bag [56], was demonstrated to be relatively successful because 70.1% of participants self-reported to use less packaging and fewer disposable shopping bags daily—the most practiced behaviour assessed in this study. Meanwhile, the Water Supplies Department organised a promotion initiative to conserve water by taking shorter showers through the Let’s Save 10L Water campaign [27], but our findings indicated nearly half of the residents never practiced nor considered showering less than five minutes every day. More should be done to investigate the awareness and promote the practice of co-benefit behaviours, especially the behaviours with low frequency in daily practice, such as buying more organic food, bringing personal eating utensils when dining in restaurants or small eateries, and having one vegetarian meal a week.

## Conclusions

This is the first study done in an Asian metropolis on the community practice of health and environmental co-benefit behaviours. The study findings indicated a great variation in the frequency of practicing co-benefit behaviours among an Asian urban population. Although over 70% of respondents reported to use less packaging and disposable shopping bags daily, four behaviours were found to have never been practiced by more than half of the population, including bringing personal eating utensils when dining in restaurants or small eateries, showering less than five minutes every day, eating one vegetarian meal a week, and buying more organic food. More advocacy and policy implementation could be carried out to encourage the practice of these co-benefit behaviours, since these behaviours provide benefits for both health and the environment.

Overall, women and older people were more inclined to practice co-benefit behaviours frequently in their daily lives, compared with men and younger people. Income and real estate ownership had mixed associations with co-benefit behaviours, since those more affluent were more likely to buy more organic food, separate household waste, and engage in active travel, but less likely to use less AC.

The demographical trends found in this study are useful for targeted promotion, especially by multidisciplinary stakeholders of the health sector, government and other civil society organizations in the community. Policy makers should incorporate concepts of co-benefits into account to ensure an optimal outcome, not only in terms of climate change and the environment, but also for the health of the population. In addition, this study provides a baseline of behaviour frequency, useful for further research on practice of co-benefit behaviours. Future research should examine if other factors (environmental policy) might be associated with the uptake of these co-benefit behaviours. Focus group study on community subgroups will also help to provide qualitative based insights to examine the potential enablers and barriers for promotion of related health programs, policies and strategies. To maximize limited resources for supporting both health protection and environmental sustainability, improving our understanding of causal pathways and predictors that might promote health and environmental co-benefit behaviours should also be a high priority for further research in the coming decades.

## Supporting information

S1 File Abridged version of climate change, extreme temperatures and health questionnaire. (PDF)
