# Supplementary material for: Socio-demographic predictors of health and environmental co-benefit behaviours for climate change mitigation in urban China
Source: PLoS One. 2017 Nov 27;12(11):e0188661. doi: 10.1371/journal.pone.0188661 (PMC5703449; doi:10.1371/journal.pone.0188661)
Supplement: S1 File — (PDF) [file pone.0188661.s001.pdf]

Climate Change, Extreme Temperatures and Health Questionnaire **Questionnaire No.** □□□□
English Abridged Version, Section: Practice of co-benefit behaviours

Date: □□Y □□M □□D Start Time: □□ : □□ Telephone No.: □□□□- □□□□
Interviewer: ______________________ Remarks: _______________

Hello Sir/Madam,
I'm an interviewer from <company name>, we are commissioned by <affiliated research institute> to conduct a survey regarding climate change, extreme temperatures, and health. May I know is there any family member who aged 15 years above? In these ___ members, who has the birthday closest to today? May I invite him/her to participate in our survey?

Hello! I'm an interviewer from <company name>, we are commissioned by <affiliated research institute> to conduct a survey regarding climate change, extreme temperatures, and health. The information collected will help to consolidate future policy on public health and medical service. The information you provided will be kept strictly confidential, will only be used for research purpose, and will not release to external parties for any purpose. If you have any enquiry, please contact <name> on <number>. Thank you for your participation.

0a) Are you willing to participate in this study?
1. □ Yes 2. □ No (Interview ends, Thank you!)

0b) Are you willing to have this interview recorded?
1. □ Yes (Start recording) 2. □ No (Continue without recording)

Part I: General health and effect of cold weather

1) Do you hold a Hong Kong ID?
1. □Yes – Permanent Hong Kong resident →Yes, continue
2. □Yes – Hong Kong resident（Domestic Helper, working or student visa holder）→Yes, continue
3. □No → Interview ends，Thank you!

2) Gender: (No need to ask if it can be identified by interviewer) 1. □Male 2. □Female

3) Do you think you are at higher health risk to extreme weather (High and low temperatures)?
1. □No 2. □Yes 888. □Don't know 999. Refuse to respond

4) What is your age? (No need to read the options, record the exact age___ years. Read the options if refuse to answer)
1. □ 15-19 2. □ 20-24 3. □ 25-29 4. □ 30-34
5. □ 35-39 6. □ 40-44 7. □ 45-49 8. □ 50-54
9. □ 55-59 10. □ 60-64 11. □ 65-69 12. □ ≥70
13. □ Refuse to respond (Interview ends)

5) How would you describe your general health (Read the options)
1. □Extremely good 2. □Very good 3. □Good 4. □Normal 5. □Bad

6) In general, how would you describe your general health currently, compared to other seasons (Read the options)

SAMPLE

1. □Much worse 2. □Worse 3. □Almost the same 4. □Better 5. □Much better

Part III: Perception and behaviors toward climate change

37) In the past 12 months, did you have the following lifestyle habits? Please indicate the frequency

		Never practiced nor considered	Never practiced but have considered	Occasionall y	At least once a week	Every day	Don’t know	Refuse to respond
a.	Use less electricity	1	2	3	4	5	888	999
b.	Use less packaging and fewer disposable shopping bags	1	2	3	4	5	888	999
c.	Use less AC	1	2	3	4	5	888	999
d.	Walk/cycle more	1	2	3	4	5	888	999
e.	Buy more organic food	1	2	3	4	5	888	999
f.	Bring personal eating utensils when dining in restaurants or small eateries	1	2	3	4	5	888	999
g.	Separate household waste	1	2	3	4	5	888	999
h.	Shower less than five minutes every day	1	2	3	4	5	888	999

38) Are you a vegetarian? 1. □ Yes 2. □ No 999. Refuse to respond

39) Do you typically have the following lifestyle habits? Please indicate the frequency

		Never practiced nor considered	Never practiced but have considered	Occasionall y	At least once a week	Every day	Don’t know	Refuse to respond
a.	Consume less meat	1	2	3	4	5	888	999
b.	Have one vegetarian meal a week	1	2	3	4	5	888	999

Part IV：Personal demographics

40) What is your education level?

1. □No formal education 2. □Primary 3. □Junior secondary 4. □Senior secondary

5. □Matriculation level 6. □Diploma 7. □Higher diploma 8. □Associate degree

9. □Bachelor 10. □Master or above 11. □Others (please indicate): ____________________

888. □Don’t know 999. □Refuse to respond

41) How much is your monthly household income (HKD)?

1. □<$2000 2. □$2000-3999 3. □$4000-5999 4. □$6000-7999

SAMPLE

5. □$8000-9999　6. □$10000-14999　7. □$15000-19999　8. □$20000-24999

9. □$25000-29999　10. □$30000-39999　11. □>$40000　888. □Don't know
999. □Refuse to respond

44) What is your marital status?
1. □Never married　2. □Cohabitation　3. □Married　4. □Separated/Divorced

5. □Widowed　6. □Others (please indicate):________　999. □Refuse to respond

45) Which district are you living?
1. □Central & Western　2. □Wan Chai　3. □Eastern　4. □Southern
5. □Yau Tsim Mong　6. □Sham Shui Po　7. □Kowloon City　8. □Wong Tai Sin
9. □Kwun Tong　10. □Kwai Tsing　11. □Tsuen Wan　12. □Tuen Mun
13. □Yuen Long　14. □North　15. □Tai Po　16. □Sha Tin
17. □Sai Kung (to 44a)　18. □Islands　888. □Don't know　999. □Refuse to respond

↓

45a) (If live in Sai Kung) Are you living in Tseung Kwan O (1. □No　2. □Yes）

46) Which type of housing are you living in?
1. □Public housing　2. □Subsidized home ownership housing　3. □Private apartments

4. □Private village house/villa　5. □Non-residential housing　6. □Temporary housing

7. □Others (please indicate): ___________　888. □Don't know　999. □Refuse to respond

47) What type of ownership do you have of your current housing?
1. □Self-owned　2. □Rental　3. □Sublessee　4. □Others (please indicate): _______

888. □Don't know　999. □Refuse to respond

== End! Thank you==

Time of end: □□ : □□

SAMPLE

Climate Change, Extreme Temperatures and Health Questionnaire
Chinese Abridged Version, Section: Practice of co-benefit behaviours

問卷號碼 ☐☐☐☐

訪問日期: ☐☐年☐☐月☐☐日　開始時間: ☐☐ : ☐☐　被訪者電話: ☐☐☐☐ - ☐☐☐☐

訪問員姓名: ____________________　覆核結果: _____________

先生／女士，你好！
我係<調查委託機構名字>嘅訪問員，我地依家受<研究機構名字>嘅委託，進行緊一項問卷調查，目的想了解民眾對氣候變化、極端氣溫和健康的認知、態度和實踐。請問你屋企依家有幾多位 15 歲或以上嘅人係度呢？_____位當中，邊一位係最近過左生日嘅呢？可以請他/ 她聽電話嗎？

你好！我係<調查委託機構名字>嘅訪問員，我地依家受香<研究機構名字>院嘅委託，進行緊一項問卷調查，目的想了解民眾對氣候變化、極端氣溫和健康認知、態度和實踐。所收集到的資料可以幫助制定更有效嘅公共衛生政策及醫療服務。而閣下提供嘅資料會絶對保密，只會作醫學研究用途，并不會向第三方作任何用途的資料披露。如有任何疑問，可致電<電話>聯絡<名字>。請你放心作答，多謝你的幫忙！

0a) 你願唔願意参加呢項研究?
1. ☐ 願意　　2. ☐ 唔願意(訪問結束，謝謝!)

0b) 你願唔願意我地將訪問過程錄音?
1. ☐ 願意 (開始錄音訪問)　　2. ☐ 唔願意 (繼續訪問, 但不要錄音!)

部分 I：健康狀況及影響

1) 你有無香港居民身份證？

4. ☐有 - 係香港永久居民 →有 ，繼續訪問
5. ☐有 - 係香港非永久性居民（外傭、持工作或讀書簽證）→ 有， 繼續訪問
6. ☐無 →訪問結束，謝謝！

2) 受訪者性別：（如已經聽出對方性別，不需问）　1. ☐男　2. ☐女

3）你覺得你在極端天氣（高溫及低溫）狀況下係唔係一個高健康風險嘅人士？
1. ☐唔係　2. ☐係　889. ☐唔知道　1000.拒答

4）你嘅年齡係？（唔要讀出選項，記錄實際年齡____歲。如果不願意答，嘗試讀出選項）

1. ☐ 15-19	2. ☐ 20-24	3. ☐ 25-29	4. ☐ 30-34
5. ☐ 35-39	6. ☐ 40-44	7. ☐ 45-49	8. ☐ 50-54
9. ☐ 55-59	10. ☐ 60-64	11. ☐ 65-69	12. ☐ ≥70
999. ☐ 拒答(終止訪問)			

5）你覺得你平時嘅健康狀況係？（請讀出選項）

SAMPLE

1. ☐極好　　2. ☐非常好　　3. ☐好　　4. ☐一般　　5. ☐差

6）一般來講，你覺得你冬季嘅健康狀況比其他季節？（請讀出選項）

1. ☐差好多　　2. ☐較差　　3. ☐差不多　　4. ☐較好　　5. ☐好好多

部分 III 對氣候變化與健康的認知及相關行為

37） 過去一年，你有無做過呢 D 生活習慣呢？（请讀出選項）

	冇做過 完全冇諗過	冇做過 － 有諗過，但冇設施/條件去做	有做過 -但好少做	有做過 － 每個禮拜都至少做一次	有做過 －日日都做	唔知道	拒答
a. 減少用電	1	2	3	4	5	888	999
b. 減少不必要包裝及購物袋	1	2	3	4	5	888	999
c. 減少開冷氣/暖氣	1	2	3	4	5	888	999
d. 多行路或踩單車	1	2	3	4	5	888	999
e. 多購買有機食品	1	2	3	4	5	888	999
f. 外出用餐自帶餐具	1	2	3	4	5	888	999
g. 家居垃圾分類	1	2	3	4	5	888	999
h. 有一個禮拜日日沖涼少過 5 分鐘	1	2	3	4	5	888	999

38） 你係唔係一個素食者？ 1. ☐係 (to 40)　　2. ☐唔係 (to 39)　　999. 拒答 （to 39）

39）你平时有無做過呢 D 生活習慣呢?（请讀出選項）

	冇做過 完全冇諗過	冇做過 － 有諗過，但冇設施/條件去做	有做過 -但好少做	有做過 － 每個禮拜都至少做一次	有做過 －日日都做	唔知道	拒答
a. 減少食肉	1	2	3	4	5	888	999
b. 一個禮拜食至少一餐素	1	2	3	4	5	888	999

部分 IV：個人資料

40) 你嘅教育程度係？

1. ☐無接受過正式教育　　2. ☐小學　　3. ☐初中　　4. ☐高中

5. ☐預科　　6. ☐文憑　　7. ☐高級文憑　　8. ☐副學士

SAMPLE

9. □學士　10. □碩士或以上　11. □其他(請註明)：__________

889. □唔知道　1000.□拒答

41) 你每月嘅家庭總收入係幾多（港幣）？

1. □<$2000　2. □$2000-3999　3. □$4000-5999　4. □$6000-7999

5. □$8000-9999　6. □$10000-14999　7. □$15000-19999　8. □$20000-24999

9. □$25000-29999　10. □$30000-39999　11. □>$40000　889. □唔知道

999. □拒答

44) 你嘅婚姻狀況係？

1. □未婚　2. □同居　3. □已婚　4. □分居／離婚

5. □喪偶　7. □其他(請註明)：__________　1000.□拒答

45) 你居住係香港邊一區?

1. □中西區　2. □灣仔　3. □東區　4. □南區
5. □油尖旺　6. □深水埗　7. □九龍城　8. □黃大仙
9. □觀塘　10. □葵青　11. □荃灣　12. □屯門
13. □元朗　14. □北區　15. □大埔　16. □沙田
17. □西貢 (需回答 44a)　18. □離島　889. □唔知道　1000.□拒答

45a) (如住在西貢區，請追問) 你係唔係住喺將軍澳？(1. □唔係　2. □係）

46) 你嘅住屋屬於邊種類型？

1. □公屋（公營租住房屋）　2. □資助自置居所房屋　3. □私人大廈/樓宇

4. □私人村屋 / 別墅　5. □非住宅用房屋　6. □臨時房屋

7. □其他(請註明)：__________　889. □唔知道　1000.□拒答

47) 你住宅租住權屬於邊種類型？

1. □自置 (有產權)　2. □全租　3. □合租/二房東/三房客　4. □其他__________

889. □唔知道　1000.□拒答

== 問卷完畢。謝謝！==

結束時間: □□:□□

SAMPLE
